# Emerging insights into plant disease management: multi-omics approaches elucidate the molecular mechanisms underlying pathogen virulence differentiation in natural populations

**DOI:** 10.1128/aem.02194-25

**Published:** 2026-01-13

**Authors:** Wenxin Song, Mi Wei

**Affiliations:** 1 School of Agriculture and Biotechnology, Shenzhen Campus of Sun Yat-Sen University 660329 , Shenzhen, Guangdong, China; Michigan State University, East Lansing, Michigan, USA

**Keywords:** rice bacterial panicle blight, multi-omics analysis, virulence differentiation, disease management

## Abstract

A recent study by R. Cheng, T. Lv, P. Ji, B. Ma, et al. (Appl Environ Microbiol 91:e01685-25, 2025, https://doi.org/10.1128/aem.01685-25) used multi-omics analysis to reveal the molecular map of pathogen virulence differentiation driven by natural variation. Building on this work, this article examines how natural variation shapes pathogen virulence and disease prevalence and explores the use of multi-omics approaches to uncover associated molecular mechanisms. The opportunities and challenges of applying multi-omics technologies in plant disease management are also discussed in this article.

## COMMENTARY

As the most important staple crop globally, rice faces numerous challenges to its secure production. Among these, bacterial panicle blight caused by *Burkholderia gladioli* represents an increasingly severe threat (1). Currently, disease management primarily relies on chemical pesticides and the cultivation of resistant varieties; however, the limitations of these approaches are becoming more evident (2). Therefore, a comprehensive understanding of the pathogen’s pathogenic mechanisms and adaptive evolutionary strategies is essential for developing precise, effective, and sustainable control measures, which is an urgent priority in ensuring rice production security. For an extended period, research on pathogen pathogenicity has predominantly focused on the identification and functional characterization of key virulence factors, such as toxins, effector proteins, and secretion systems (3). These studies typically involve comparisons between laboratory-generated virulence mutants or among species and strains exhibiting marked differences in virulence. However, pathogen populations are neither uniform nor static; rather, they exhibit substantial genetic diversity. In natural environments, pathogens have continuously adapted to diverse biotic (e.g., host resistance, microbial interactions) and abiotic (e.g., climate conditions, agricultural practices) selective pressures, leading to naturally occurring genetic variations. Although these variations may be subtle, they can profoundly influence pathogen fitness, transmissibility, and virulence (4). A comprehensive analysis of such fine-scale genetic differences, combined with an understanding of the molecular mechanisms underlying virulence divergence from the perspective of adaptive evolution, is essential for predicting disease outbreaks and refining disease control strategies. Advances in omics sequencing technologies have provided comprehensive insights into plant pathogens across multiple levels of biological activity, including genomic composition, gene transcription, and protein expression (5). However, prior research has predominantly emphasized the analysis of single-omics data and subsequent validation of molecular mechanisms, thereby overlooking the integrative process spanning from “genetic information interpretation” to the “functional execution” underlying pathogen life activities (6). Cheng et al.’s study, through an integrated comparative genomics and transcriptomics analysis of two *B. gladioli* strains, ZJ-SD (highly virulent) and ZJ-MD (low virulence), systematically elucidated the molecular mechanisms underlying virulence differentiation in the rice panicle blight pathogen *B. gladioli* under conditions of natural variation. This work represents a conceptual advancement from localized mechanisms to comprehensive regulatory networks, offering a robust model for multi-level and systematic understanding of pathogen virulence differentiation (7).

## THE NATURAL VARIATION OF PATHOGENS IS THE CORE FACTOR IN THE PREVALENCE OF PLANT DISEASES

Traditional plant pathology research has predominantly focused on analyzing the pathogenic mechanisms of highly virulent and prevalent strains, as well as the genetic variation underlying pathogenicity in laboratory-induced mutant strains. However, it has largely overlooked the adaptive evolution of pathogens and the resulting mechanisms of virulence differentiation (8). Plant pathogens, including fungi, oomycetes, bacteria, and viruses, are not static entities but dynamic populations characterized by high genetic plasticity. Their ongoing variation and evolution constitute the primary drivers behind the emergence of novel diseases, the resurgence of previously controlled ones, and the failure of established control measures, such as resistant crop varieties and chemical fungicides. This process constitutes an evolutionary cycle driven by genetic diversity within pathogen populations, environmental selection pressures, and fitness trade-offs (9).

Genetic variation within pathogen populations primarily arises through mechanisms including gene mutation, genetic recombination via sexual reproduction, parasexuality, and horizontal gene transfer, as well as genomic alterations such as transposon activity, chromosomal segment duplication, or deletion. These processes collectively contribute to the preservation and dissemination of genetic diversity among pathogen populations, thereby laying the groundwork for adaptive evolution under selective pressure (10). For instance, genomic single nucleotide polymorphisms (SNPs), insertions and deletions (INDELs), and structural variations (SVs) can directly cause the differentiation of pathogen virulence. In the study by Chen et al., *B. gladioli* accumulated 79 SNPs, 12 INDELs, and 3 SVs, ultimately leading to significant differentiation in virulence-related phenotypes such as biofilm formation and motility, thereby causing differences in the field damage of bacterial panicle blight in rice (7).

Environmental selection pressures primarily encompass the overuse of pesticides, monoculture of disease-resistant crop varieties, continuous cultivation of single crops, and abnormal climatic conditions. Pesticides, acting as chemical mutagens, can directly damage pathogen DNA and elevate gene mutation rates (11). Disease-resistant plant varieties activate immune responses through the recognition of pathogen effectors. Pathogens harboring genetic variations in effector genes can evolve into “escape” variants that evade host immune defenses by avoiding detection and recognition by the host immune system (12). Abnormal environmental conditions such as extreme temperatures and drought can activate stress response pathways in pathogens, increase errors in DNA repair process, and consequently enhance the frequency of genetic mutations. Additionally, climate warming may expand the geographic distribution of pathogens; when geographically distinct populations come into contact, genetic recombination can occur, leading to novel genetic variants (13). Continuous monoculture significantly increases pathogen population density and the frequency of inter-individual interactions, facilitating horizontal gene transfer and accelerating both the dissemination and emergence of genetic variations (14). In addition, agricultural operations (such as irrigation and mechanical farming) carry different pathogen populations, promoting gene exchange among populations and, thus, generating new variations.

Natural selection pressure can also create new adaptive niches, thereby enhancing the adaptability and survival capabilities of certain mutant strains. Under continuous selection pressure, mutant strains possessing fitness advantages exhibit a significantly higher reproduction rate compared to ordinary strains, and their proportion within the population gradually increases (15). Through agricultural operations, air currents, insect vectors, and other means, mutant strains are capable of spreading across regions, thus expanding the distribution range of the mutations within the entire pathogen population. Pathogens are capable of directly transmitting genetic variations generated via gene mutation or recombination to their progeny through asexual reproduction (e.g., binary fission in bacteria and spore reproduction in fungi) or sexual reproduction, thereby attaining stable inheritance of the variations (16). The variant genes carried by mobile genetic elements, such as drug-resistance plasmids and transposons, can be transferred among pathogen individuals across different generations, overcoming the inter-generational limitations of sexual reproduction and facilitating the accumulation and spread of variant genes across generations. Beneficial variations in pathogens are continuously retained under selective pressure and gradually become the dominant genotypes within the population through multiple generations of reproduction, resulting in the formation of a stably inherited variant population (17).

Currently, strategies such as intercropping disease-resistant varieties, implementing crop rotation, and rotating fungicide applications are commonly employed to slow the adaptive evolution of pathogens. These approaches aim to reduce selection pressure and prolong the effectiveness of disease control measures (18). However, their success has been limited, and they remain insufficient in fundamentally mitigating pathogen virulence differentiation and the emergence of resistance. Therefore, a comprehensive investigation into the molecular mechanisms underlying pathogen evolution is essential for achieving precise and sustainable disease control.

## MULTI-OMICS TECHNOLOGIES OFFER CRUCIAL CLUES FOR CLARIFYING THE MOLECULAR MECHANISMS UNDERLYING PATHOGEN VIRULENCE DIFFERENTIATION

Single-omics approaches are inherently limited in elucidating the molecular mechanisms that govern the entire spectrum of life activities from genetic basis and expression regulation to functional execution and phenotypic output. In contrast, the integrated application of multi-omics technologies, including genomics, transcriptomics, proteomics, and metabolomics, enables a comprehensive exploration of molecular dynamics through stepwise molecular profiling and cross-layer data integration (19). Specifically, genomics identifies genetic variations, transcriptomics captures gene expression patterns, proteomics delineates protein-level functional activities, and metabolomics reflects physiological and metabolic alterations. By integrating evidence from multi-omics data sets, researchers can formulate key hypotheses regarding molecular mechanisms, which are subsequently validated through targeted genetic experiments such as gene knockout, RNA interference, and overexpression. This systematic framework, linking molecular insights from genetic information decoding to the execution of biological functions, offers a more holistic and rigorous understanding of the molecular basis of pathogen virulence differentiation, thereby establishing a solid theoretical foundation for identifying precise targets for disease prevention and control. In the study by Cheng et al., variant genes in *B. gladioli* were mapped to virulence-associated pathways, including quorum sensing, cationic antimicrobial peptide resistance, and histidine metabolism. Protein domain prediction revealed that the mutant gene is significantly associated with the synthesis of key protein factors, including alginate export proteins involved in biofilm formation, helix-turn-helix (HTH) domain-containing transcription factors implicated in gene regulation, and IS3 family transposases potentially driving genomic rearrangements. Notably, these mutant genes are not randomly distributed but are predominantly clustered within a “virulence regulatory network.” These results indicate that natural genetic variation contributes to virulence differentiation by directly altering the function or stability of specific pathogen proteins or by modulating the activity of regulatory elements. Transcriptomic analysis further elucidated the mechanistic link between genetic variation and phenotypic divergence: differentially expressed genes (DEGs) were significantly enriched in core virulence pathways including two-component systems, bacterial chemotaxis, and flagellar assembly, findings that align with both genomic variation and observed changes in virulence-related phenotypes such as flagellar production, quorum sensing, chemotaxis, and biofilm formation (7).

It is worth noting that the advancement of multi-omics technologies must move beyond the limitations of single-object studies and evolve toward cross-scale integrated analysis of the “pathogen–host–environment” system (20). By simultaneously characterizing the omics profiles associated with pathogen virulence, the molecular profiles of host immune responses, and the mechanisms of environmental factor influences, the external driving factors and internal regulatory pathways of virulence differentiation can be fully revealed. Such cross-scale analysis not only uncovers the core genetic determinants, regulatory pathways, and environmental drivers of virulence variation but also provides precise molecular targets for the development of targeted inhibitors and the screening of biocontrol agents, thereby promoting the transition of agricultural disease management toward precision and sustainability.

## OPPORTUNITIES AND CHALLENGES OF MULTI-OMICS TECHNOLOGIES IN THE MANAGEMENT OF PLANT DISEASES

Multi-omics technology has emerged as a highly promising approach for elucidating the mechanisms underlying pathogen virulence differentiation. However, conventional bulk sequencing methods, which analyze thousands of cells collectively, limit the ability to capture cellular-level molecular heterogeneity. The advent of advanced sequencing technologies, such as single-cell sequencing and spatial transcriptomics, has enabled more in-depth and multidimensional insights into the molecular mechanisms governing virulence (21, 22). Single-cell sequencing enables the precise characterization of gene expression at the individual cell level. In pathogen populations occupying the same ecological niche, this approach reveals heterogeneity in the expression of virulence-related genes across cells, thereby enabling more accurate discrimination between highly virulent and low-virulence subpopulations. By integrating single-cell data from the early, middle, and late stages of infection, it is possible to reconstruct a dynamic, high-resolution map of virulence gene expression, facilitating the identification of novel genes that are critically involved in specific phases of the infection process. Moreover, single-cell sequencing allows for continuous monitoring of gene expression across key developmental transitions, such as spore germination, appressorium formation, and hyphal expansion, offering mechanistic insights into the initiation and regulation of pathogenic virulence throughout the life cycle (23).

Spatial transcriptomics technology enables the simultaneous acquisition of gene expression profiles and their precise spatial context within host tissues, thereby facilitating a more comprehensive understanding of the spatially organized dynamics of pathogen infection in plants (24). By applying this approach, the expression patterns of pathogen virulence genes and plant defense-related genes can be precisely resolved across distinct anatomical regions of plant lesions, including the necrotic center, the junction between lesion and healthy tissue, and the infection front, providing spatially resolved insights into host–pathogen interactions. Moreover, analysis of spatial transcriptomics data enables the resolution of pathogen preferences for specific plant cell types during colonization and infection, thereby providing insights into the pathogen’s host targeting strategies. Notably, plant defense responses exhibit distinct spatial specificity. Spatial transcriptomics enables the identification of key tissue sites involved in the response to pathogen infection and reveals the spatial patterns of immune signal transmission within plant tissues.

Currently, artificial intelligence technology has exhibited distinctive advantages in high-dimensional data dimensionality reduction, core target screening, and regulatory network prediction. It serves as a crucial tool for in-depth analysis of multi-omics data (25). Artificial intelligence algorithms are capable of projecting complex multi-dimensional data into low-dimensional spaces, thus enabling the visualization of different cell clusters or spatial regions. Through the analysis of co-expression relationships among genes, artificial intelligence technology can assist in identifying the key transcription factors that regulate the expression of virulence genes and constructing a regulatory network governing the core genes of virulence differentiation. Artificial intelligence models can also integrate and analyze multi-omics sequencing data, such as single-cell transcriptomics and spatial transcriptomics, to construct a more comprehensive spatiotemporal map of virulence regulation. Based on the current understanding of pathogenic molecular mechanisms, targeted artificial intelligence models can be trained to predict the potential functions of new pathogenic genes of pathogenic bacteria and conducting rapid diagnosis of the virulence level or evolutionary stage of pathogenic bacteria according to the gene expression profile (26).

The development and application of multi-omics technologies face several key constraints: substantial heterogeneity across diverse omics data, lack of standardized data analysis protocols, challenges in high-throughput validation of candidate molecular targets, and high demands for experimental costs and computational resources (27).

Compared to studies focused on humans or other animals, the application of multi-omics sequencing technology in elucidating the pathogenic mechanisms and virulence differentiation of plant pathogenic bacteria encounters multiple challenges. First, plant–pathogen interactions are highly susceptible to environmental factors, and plants lack an adaptive immune system, relying primarily on cell-autonomous innate immunity and systemic signal transduction. This results in limited experimental controllability and increased complexity in data interpretation (28). Second, in plant pathology research, it is particularly challenging to obtain single-cell suspensions containing intact plant and pathogen cells. Consequently, transcriptomic or proteomic analyses of pathogens are often confounded by substantial host-derived signal contamination, placing high demands on the sensitivity of detection methods. More critically, effective statistical tools for accurately distinguishing pathogen-derived signals from host signals are still lacking, which significantly hinders downstream molecular mechanism studies. Additionally, the genetic transformation efficiency of numerous significant plant pathogens (particularly fungi and oomycetes) is remarkably low, and effective gene-editing systems are often unavailable. This situation presents a substantial challenge to the functional validation of candidate molecular targets (29). Ultimately, the genomic diversity of plant pathogens is exceedingly high, with notable disparities in size and structure. Furthermore, the reference genomes of many species are incompletely assembled and inadequately annotated. Notably, the genomic regions abundant in repetitive sequences are frequently the “blind spots” of genome assembly, while they are the crucial genetic targets for virulence differentiation (30). Moreover, the establishment of large-scale, diverse, and shared databases remains inadequate in plant pathology research, resulting in insufficient data support for integrated analysis and artificial intelligence model training.

In the future, it will be essential to establish standardized experimental and analytical protocols. By integrating high-throughput mutant screening and protein interaction technologies, a closed-loop research system of “multi-omics prediction-high-throughput validation” ought to be constructed. Through the establishment of a globally shared pathogen multi-omics database, the generalization ability of artificial intelligence models can be enhanced, and more efficient cross-omics integration mathematical models can be developed. As these challenges are gradually addressed, multi-omics technology will play a more significant role in the study of pathogen virulence differentiation, providing a robust theoretical and technical foundation for achieving sustainable and precise disease control.

## References

[1] Protic IA, Uddin MN, Tushar ASM, Auyon ST, Alvarez-Ponce D, Islam MR. 2024. First complete genome sequence of a bacterial panicle blight causing pathogen, Burkholderia glumae, isolated from symptomatic rice grains from Bangladesh. BMC Genom Data 25:73. doi:10.1186/s12863-024-01255-5 39075351 PMC11287961

[2] Zheng X, Peng Y, Qiao J, Henry R, Qian Q. 2024. Wild rice: unlocking the future of rice breeding. Plant Biotechnol J 22:3218–3226. doi:10.1111/pbi.14443 39150344 PMC11501002

[3] Li J, Ai M, Hou J, Zhu P, Cui X, Yang Q. 2024. Plant–pathogen interaction with root rot of Panax notoginseng as a model: insight into pathogen pathogenesis, plant defence response and biological control. Mol Plant Pathol 25:1–15. doi:10.1111/mpp.13427

[4] Sato Y, Bex R, van den Berg GCM, Santhanam P, Höfte M, Seidl MF, Thomma BPHJ. 2025. Starship giant transposons dominate plastic genomic regions in a fungal plant pathogen and drive virulence evolution. Nat Commun 16:6806. doi:10.1038/s41467-025-61986-6 40707455 PMC12289983

[5] Wang X, Sun Q, Liu T, Lu H, Lin X, Wang W, Liu Y, Huang Y, Huang G, Sun H, Chen Q, Wang J, Tian D, Yuan F, Liu L, Wang B, Gu Y, Liu B, Chen L. 2024. Single-cell multi-omics sequencing uncovers region-specific plasticity of glioblastoma for complementary therapeutic targeting. Sci Adv 10:4306–4312. doi:10.1126/sciadv.adn4306 PMC11584018 39576855

[6] Ji Q, Jiang X, Wang M, Xin Z, Zhang W, Qu J, Liu G-H. 2024. Multimodal omics approaches to aging and age-related diseases. Phenomics 4:56–71. doi:10.1007/s43657-023-00125-x 38605908 PMC11003952

[7] Cheng R, Lv T, Ji P, Ma B, Wang M, Matsumoto H. 2025. Multi-omics analyses reveal the virulence differentiation underlying natural variation in Burkholderia gladioli. Appl Environ Microbiol 91:e01685-25. doi:10.1128/aem.01685-25 41258717 PMC12724388

[8] Saha B, Nayak J, Srivastava R, Samal S, Kumar D, Chanwala J, Dey N, Giri MK. 2023. Unraveling the involvement of WRKY TFs in regulating plant disease defense signaling. Planta 259:1–26. doi:10.1007/s00425-023-04269-y 38012461

[9] Yu Z, Coorens THH, Uddin MM, Ardlie KG, Lennon N, Natarajan P. 2024. Genetic variation across and within individuals. Nat Rev Genet 25:548–562. doi:10.1038/s41576-024-00709-x 38548833 PMC11457401

[10] Dodds PN. 2010. Plant science. Genome evolution in plant pathogens. Science 330:1486–1487. doi:10.1126/science.1200245 21148378 PMC3076603

[11] Zhou W, Li M, Achal V. 2025. A comprehensive review on environmental and human health impacts of chemical pesticide usage. Emerg Contam 11:100410. doi:10.1016/j.emcon.2024.100410

[12] Li Q, Zhu H, Ai G, Yu J, Dou D. 2024. Plant genes related to Phytophthora pathogens resistance. Phytopathol Res 6:1–11. doi:10.1186/s42483-024-00229-w

[13] Lahlali R, Taoussi M, Laasli S-E, Gachara G, Ezzouggari R, Belabess Z, Aberkani K, Assouguem A, Meddich A, El Jarroudi M, Barka EA. 2024. Effects of climate change on plant pathogens and host-pathogen interactions. Crop Environ 3:159–170. doi:10.1016/j.crope.2024.05.003

[14] Roussin-Léveillée C, Rossi CAM, Castroverde CDM, Moffett P. 2024. The plant disease triangle facing climate change: a molecular perspective. Trends Plant Sci 29:895–914. doi:10.1016/j.tplants.2024.03.004 38580544

[15] Schäfer W. 1994. Molecular mechanisms of fungal pathogenicity to plants. Annu Rev Phytopathol 32:461–477. doi:10.1146/annurev.py.32.090194.002333

[16] Hovmøller MS, Thach T, Justesen AF. 2023. Global dispersal and diversity of rust fungi in the context of plant health. Curr Opin Microbiol 71:102243. doi:10.1016/j.mib.2022.102243 36462410

[17] Islam T, Haque MA, Barai HR, Istiaq A, Kim J-J. 2024. Antibiotic resistance in plant pathogenic bacteria: recent data and environmental impact of unchecked use and the potential of biocontrol agents as an eco-friendly alternative. Plants (Basel) 13:1135–1167. doi:10.3390/plants13081135 38674544 PMC11054394

[18] Omaye JD, Ogbuju E, Ataguba G, Jaiyeoba O, Aneke J, Oladipo F. 2024. Cross-comparative review of machine learning for plant disease detection: apple, cassava, cotton and potato plants. Artif Intell Agric 12:127–151. doi:10.1016/j.aiia.2024.04.002

[19] Chen S, Wang Z. 2024. Integration of mult-omics and nucleotide metabolism reprogramming signature analysis reveals gastric cancer immunological and prognostic features. Cancer Cell Int 24:212. doi:10.1186/s12935-024-03396-0 38880869 PMC11180389

[20] Ballard JL, Wang Z, Li W, Shen L, Long Q. 2024. Deep learning-based approaches for multi-omics data integration and analysis. BioData Min 17:38. doi:10.1186/s13040-024-00391-z 39358793 PMC11446004

[21] Wu X, Yang X, Dai Y, Zhao Z, Zhu J, Guo H, Yang R. 2024. Single-cell sequencing to multi-omics: technologies and applications. Biomark Res 12:110. doi:10.1186/s40364-024-00643-4 39334490 PMC11438019

[22] Jin Y, Zuo Y, Li G, Liu W, Pan Y, Fan T, Fu X, Yao X, Peng Y. 2024. Advances in spatial transcriptomics and its applications in cancer research. Mol Cancer 23:129. doi:10.1186/s12943-024-02040-9 38902727 PMC11188176

[23] Jovic D, Liang X, Zeng H, Lin L, Xu F, Luo Y. 2022. Single-cell RNA sequencing technologies and applications: a brief overview. Clin Transl Med 12:e694. doi:10.1002/ctm2.694 35352511 PMC8964935

[24] Tian L, Chen F, Macosko EZ. 2023. The expanding vistas of spatial transcriptomics. Nat Biotechnol 41:773–782. doi:10.1038/s41587-022-01448-2 36192637 PMC10091579

[25] Zhang K, Yang X, Wang Y, Yu Y, Huang N, Li G, Li X, Wu JC, Yang S. 2025. Artificial intelligence in drug development. Nat Med 31:45–59. doi:10.1038/s41591-024-03434-4 39833407

[26] Bock CH, Barbedo JGA, Mahlein A-K, Del Ponte EM. 2022. A special issue on phytopathometry — visual assessment, remote sensing, and artificial intelligence in the twenty-first century. Trop plant pathol 47:1–4. doi:10.1007/s40858-022-00498-w

[27] Wang X, Fan D, Yang Y, Gimple RC, Zhou S. 2023. Integrative multi-omics approaches to explore immune cell functions: challenges and opportunities. iScience 26:106359. doi:10.1016/j.isci.2023.106359 37009227 PMC10060681

[28] Jones JDG, Staskawicz BJ, Dangl JL. 2024. The plant immune system: from discovery to deployment. Cell 187:2095–2116. doi:10.1016/j.cell.2024.03.045 38670067

[29] Gorshkov V, Tsers I. 2022. Plant susceptible responses: the underestimated side of plant-pathogen interactions. Biol Rev Camb Philos Soc 97:45–66. doi:10.1111/brv.12789 34435443 PMC9291929

[30] Sun C, Lei Y, Li B, Gao Q, Li Y, Cao W, Yang C, Li H, Wang Z, Li Y, Wang Y, Liu J, Zhao KT, Gao C. 2024. Precise integration of large DNA sequences in plant genomes using PrimeRoot editors. Nat Biotechnol 42:316–327. doi:10.1038/s41587-023-01769-w 37095350

